# Oxytocin administration during labor does not affect maternal serum oxytocin levels, early postpartum breastfeeding attitude and maternal attachment

**DOI:** 10.1371/journal.pone.0337762

**Published:** 2025-12-05

**Authors:** Meryem Özdemir, Esin Çeber Turfan, Hüseyin Levent Keskin

**Affiliations:** 1 Delivery Unit, Ankara City Hospital, Ankara, Türkiye; 2 Department of Midwifery, Institute of Health Sciences, Ege University, İzmir, Türkiye; 3 Department of Midwifery, Faculty of Health Sciences, Ege University, İzmir, Türkiye; 4 Obstetrics and Gynecology Clinic, Ankara City Hospital, Ankara, Türkiye; 5 Department of Obstetrics and Gynecology, Ufuk University School of Medicine, Ankara, Türkiye; Ankara University Faculty of Nursing, TÜRKIYE

## Abstract

The goal was to investigate the effects of synthetic oxytocin administration during labor on maternal and neonatal serum oxytocin levels, early breastfeeding attitudes, and postpartum maternal attachment immediately after birth. This prospective case-control study was carried out in the delivery unit of a tertiary hospital. The pregnant women who were at term and had an uncomplicated low-risk single pregnancy were enrolled in this study. The cases that required receiving i.v. synthetic oxytocin during labor constituted the study group, who didn’t receive synthetic oxytocin were included in the control group. From the cases who gave birth vaginally and met criteria, maternal venous and umbilical cord blood samples were taken immediately after delivery. Serum oxytocin test analysis was performed using the Competitive ELISA method. The socio-demographic characteristics, obstetric history of mothers, and neonatal features were recorded from the medical records. Early breastfeeding attitude of mothers was assessed using the Breastfeeding Attitudes Evaluation Scale (BAES). The Maternal Attachment Scale (MAS) was used to evaluate the experiences, feelings, and emotions of mothers. *p-value* <0.05 was accepted as statistically significant. There is no difference between the age, other socio-demographic, clinical, and obstetric data of mothers in the study group (n = 75) and the control group (n = 78) (p > 0.05), except for the time to hold the baby for the first time (p = 0.016). The maternal serum mean oxytocin levels of the maternal and umbilical cord blood plasma oxytocin levels of their newborns were similar between the groups (p = 0.331, p = 0.475; respectively). No statistically significant difference was observed between the groups in the BAES scores and the MAS scores (p > 0.05). It was observed that intravenous synthetic administration during labor did not affect the maternal and neonatal serum oxytocin levels and had no effect on early breastfeeding attitude and maternal attachment.

## Introduction

Oxytocin is the main hormone that plays an active role during labor, the postpartum period, and breastfeeding. In addition to its obstetric effects, it plays a role in many physiological, emotional, and behavioral processes of the organism, such as maternal attachment, maternal-fetal bonding, lactation, empathy, trust, and stress regulation [1,2]. Synthetic oxytocin is the most commonly used pharmacologic agent to induce or augment labor when used discreetly when there are indications, and has vitally important beneficial effects such as postpartum hemorrhage prophylaxis and treatment [3]. Synthetic oxytocin administration has potential side effects such as uterine tachysystole and fetal distress that may develop due to uterine contractility [4]. Although synthetic oxytocin shares the same chemical structure as endogenous oxytocin, its relationship with endogenous oxytocin is unclear [5,6]. The endogenous oxytocin released during the labor and delivery process plays important roles not only for birth itself but also for preparing for adaptations that affect breastfeeding and maternal well-being in the postpartum period [7]. However, it has been reported that this balanced endogenous oxytocin system is affected by the administration of synthetic oxytocin during the intrapartum period [5]. Besides the serious side effects, the administration of synthetic oxytocin during labor may affect the endogenous oxytocin system and, as a result, influence maternal bonding, stress, and maternal behaviors, including lactation and breastfeeding. Synthetic oxytocin administered during the intrapartum period was associated with the newborn’s instinctive sucking reflex, and the relationship of synthetic oxytocin with sucking and breastfeeding was dose-dependent [8]. This can lead to difficulties in breastfeeding as it negatively affects the maternal attachment that develops between the mother and baby.

In the literature on oxytocin, the most commonly focused topics are its role in various maternal physiological processes and behaviors, including childbirth, breastfeeding, maternal well-being, maternal adaptation, and mother-infant bonding [9,10]. However, it has been observed that studies generally examine oxytocin levels in response to the development of maternal physiological processes or behaviors, and whether these levels are affected by synthetic oxytocin [11].

We hypothesized that maternal serum oxytocin levels would show significant differences between the group receiving synthetic oxytocin and those not receiving it. The main goal of this study was to investigate the effects of synthetic oxytocin administered to initiate or support labor on maternal and neonatal serum oxytocin levels, early breastfeeding attitudes, and postpartum maternal attachment by measuring maternal and neonatal serum oxytocin levels immediately after birth.

## Materials and methods

This prospective case-control study was carried out in the delivery unit of a tertiary hospital between January 2020 and February 2021. The ethical approval was obtained from Local Ethics Committee No.1 (Ethics Approval No: E1-20–965).

The pregnant women, who were at term (between 38^th^ and 41^st^ week of gestation), bringing a single healthy fetus, have an uncomplicated low-risk pregnancy and have no chronic disease, were enrolled in this study. The cases that required receiving i.v. synthetic oxytocin for the induction or augmentation of labor constituted the study group. The decision to administer synthetic oxytocin was made by the obstetrician. The low-dose oxytocin regimen was used to administer synthetic oxytocin. The exogenous oxytocin solution was prepared as 10 U in 1000 mL saline solution. The initial administration rate of the prepared solution was 12 ml/h for all participants. The infusion rate was generally increased by 12 ml/h every 15 min until 3–4 contractions lasting at least 40 s occurred within 10 min. The maximum rate administered was 48 ml/h, equivalent to a dosage rate of 10 mIU/min. The synthetic oxytocin dosage regimen did not change, but the total dose administered varied greatly. The mean total volume of exogenous oxytocin solution (10 IU/L) administered was 166 ± 168 ml (ranged from 9 ml to 936 ml), and the mean length of administration was 3.8 ± 3.7 hours (ranged from 30 minutes to 19 hours).

The cases that didn’t receive synthetic oxytocin during labor were included in the control group. For both groups the mothers, who had high-risk pregnancies for mother and fetus, required emergency cesarean section due to any indication, required any intervention during delivery (e.g., shoulder dystocia or operative delivery), separated from the baby for neonatal care for any reason after delivery, had unwillingness to breastfeed her baby, and had any health problem in the herself or her baby that would prevent breastfeeding were not enrolled in the study. Considering that the half-life of synthetic oxytocin is very short (2–4 minutes), exogenous oxytocin was administered to all cases included in the study until birth occurred.

All participants had given written informed consent.

The G*Power 3.1.9.7 program was used to calculate the sample size for two groups. It was found that a sample size of at least 150 individuals (75 subjects for each group) was sufficient with a 95% confidence interval. Studies examining oxytocin levels were considered in determining the sample size [12].

From the cases who gave birth vaginally and met the inclusion and exclusion criteria, 3–5 mL maternal venous blood samples were taken immediately after delivery from 75 mothers in the study group and 78 mothers in the control group.

Umbilical cord clamping was performed at least 30 seconds after delivery. After skin-to-skin contact was provided immediately, routine care was given to the newborn. In both groups of all newborns, about 4–5 mL of the umbilical cord blood sample was taken from the cord section remaining on the maternal side.

The serum of the blood samples were kept at −80 °C. The ELABSCIENCE^®^ 96-well plate commercial Oxytocin EIA kit with the product number E-EL-0029 (R&D Systems, USA) was used to determine the serum oxytocin levels. The kit only recognizes oxytocin, not other nonapeptides such as vasopressin. Serum oxytocin test analysis was performed using the Competitive ELISA method.

The socio-demographic characteristics, obstetric history of mothers, and neonatal features were recorded from the medical records. The participants were questioned about early breastfeeding and attachment attitudes in the postpartum period. Breastfeeding Attitudes Evaluation Scale (BAES), including 22 positive and 24 negative items about breastfeeding attitudes, was administered to the subjects to evaluate whether mothers had a positive or negative attitude towards breastfeeding [13]. The total score of the scale is 184, of which 88 are positive items and 96 are negative items. Evaluation is made according to the total score obtained from all items, and the attitude of the mothers towards breastfeeding is evaluated positively as the score obtained from the scale increases.

Maternal Attachment Scale (MAS) was used to evaluate the experiences, feelings, and emotions of mothers [14]. The lowest score obtainable from the scale is 26, and the highest score is 104. As the score obtained from the scale increases, it is interpreted as maternal attachment being positive and tight.

The mothers spent the first two hours of the postpartum period in the delivery room, and scale forms were completed in this period before the mothers had their first breastfeeding experience, and before being transferred to the postpartum services.

The statistical analysis of the data was done using SPSS ver. 27.0 software. The descriptive values were shown as mean ±SD, median (minimum – maximum), number, and percentages. The Student T-test was used to compare the normally distributed continuous variables, and the Mann-Whitney test was used for the data without a normal distribution. The Chi-square test was used to compare categorical variables. *p-value* <0.05 was accepted as statistically significant.

## Results

A total of 153 mothers and 153 their newborns (case group n = 75, control group n = 78) participated in the study.

A total of 306 blood samples were analyzed to determine the serum oxytocin level.

There is no difference between the mean ages of mothers in the study group (received synthetic oxytocin during labor) and the control groups (26.9 ± 5.4 *vs* 26.5 ± 5.7, p = 0.569). Other socio-demographic, clinical, and obstetric data were also similar between the two groups (p > 0.05) except for the time to hold the baby for the first time (41 ± 33 *vs* 27 ± 16, p = 0.016) (Table 1).

**Table 1:** The characteristics of subjects who received synthetic oxytocin (study group) and who didn’t (control group).

		Study Group (n = 75)	Control Group (n = 78)	p
Maternal age (years)	Mean ±SD (min – max)	26.9 ± 5.4 (18 - 38)	26.5 ± 5.7 (16–39)	0.569
Paternal age (years)		31.3 ± 6.9 (21 - 49)	30.3 ± 6.6 (21-45)	0.312
Duration of marriage (years)		6.1 ± 4.4 (1 - 22)	5.6 ± 3.9 (1 - 20)	0.787
Parity (n) (median (min – max))		0 (0–3)	0 (0–4)	0.737
Education level (n; %)	≤8 years	32 (42.7)	29 (37.2)	0.462
	8-12 years	32 (42.7)	42 (53.8)	
	>12 years	11 (14.7)	7 (9.0)	
Employment (n; %)	Yes	7 (9.3)	13 (16.7)	0.615
	No	68 (90.7)	65 (83.3)	
Income level (n; %)	High	21 (28.8)	32 (41.0)	0.198
	Medium	41 (56.2)	33 (42.3)	
	Low	11 (15.1)	13 (16.7)	
Intended pregnancy (n; %)	Yes	62 (82.7)	60 (76.9)	0.495
	No	13 (17.3)	18 (23.1)	
Desired gender of baby (n; %)	Yes	71 (94.7)	69 (88.5)	0.277
	No	4 (5.3)	9 (11.5)	
Receiving antenatal care (n; %)	Yes	61 (81.3)	64 (82.1)	1.000
	No	14 (18.7)	14 (18.0)	
Birth weight (g) (Mean ±SD (min – max))		3311 ± 348 (2730–4200)	3424 ± 361 (2460–4250)	0.926
Apgar score (1^st^ min) (n; %)	<7	10 (13.3)	8 (10.3)	0.366
	≥7	65 (86.7)	70 (89.7)	
Apgar score (5^th^ min) (n; %)	<7	–	–	NA
	≥7	75 (100)	78 (100)	
Time to want to see her baby for the first time (min)	Median (min – max)	1 (1-60)	1 (1-60)	0.066
Time to hold her baby for the first time (min)		41 ± 33 (12 - 300)	27 ± 16 (10 - 180)	0.016
Time to breastfeed for the first time (min)		48 ± 16 (6–80)	40 ± 11 (4–70)	0.139

The maternal serum mean oxytocin levels of the mothers (1424 ± 213 pg/mL in the cases *vs* 1366 ± 253 pg/mL in the controls), and umbilical cord blood plasma oxytocin levels of their newborns were similar between the groups (1418 ± 165 pg/mL *vs* 1381 ± 196 pg/mL) (p = 0.331, p = 0.475; respectively) (Table 2).

**Table 2:** Maternal and umbilical cord blood serum plasma oxytocin levels, Breastfeeding Attitudes Evaluation Scale (BAES) scores, Maternal Attachment Scale (MAS) scores of mothers who received synthetic oxytocin (study group) and those who did not (control group).

	Study Group (n = 75)	Control Group (n = 78)	p
	Mean ±SD (min – max)		
Maternal blood serum synthetic oxytocin level (pg/mL)	1424 ± 213 (413–2000)	1366 ± 253 (274–1818)	0.331
Umbilical cord blood serum synthetic oxytocin level (pg/mL)	1418 ± 165 (1000–1914)	1381 ± 196 (500 −1714)	0.475
BAES score	110.8 ± 12.7 (64–144)	111.0 ± 12.7 (68–136)	0.922
MAS score	100.9 ± 3.4 (89–104)	98.9 ± 5.8 (78–104)	0.545

No statistically significant difference was observed between the cases administered oxytocin during labor (study group) and the control group (cases not administered oxytocin) in the BAES scores applied to evaluate breastfeeding status (p = 0.922) and the MAS scores applied to evaluate mother-baby attachment status (p = 0.545) (Table 2).

## Discusssion

There is no definite norm value range for gestational and postpartum oxytocin levels, because oxytocin levels were measured at different times and later in the postpartum period in the literature. In addition, the application of different interventions during labor that may affect the oxytocin level may have caused these differences. Therefore, it is not possible to consider whether the oxytocin levels measured in this study were within normal ranges. Most factors, such as pregnancy, labor, breastfeeding, the uterine involution process, the mother-infant relationship in the early postpartum period, stress, sexual stimulation, and social interactions affect oxytocin levels [15–17]. In addition to these factors, some studies have shown that synthetic oxytocin administration during labor also affects oxytocin levels [16,18]. Peripherally administered synthetic oxytocin can only increase oxytocin levels in the peripheral system because it crosses the blood-brain barrier to a limited extent. This may cause negative feedback to the central nervous system, leading to a decrease in endogenous oxytocin release to the peripheral system [6]. A positive association was found between synthetic oxytocin administration during the intrapartum period and endogenous oxytocin levels measured in the postpartum period [19]. This suggests that synthetic oxytocin administered during labor can modulate women’s oxytocin system in the long term, but these studies have reported conflicting results between endogenous oxytocin levels and breastfeeding [19]. The pulsatile pattern of endogenous oxytocin release may also explain the differences between studies depending on when the blood was drawn [10,20]. In our study, oxytocin was administered until birth occurred, and blood samples were taken immediately afterwards. A recent study reported that intrapartum synthetic oxytocin infusion increased maternal plasma oxytocin levels in a dose-dependent manner, with doubling the infusion rate approximately doubling plasma oxytocin levels [21]. This study mainly investigated whether synthetic oxytocin administered for labor induction or augmentation affected maternal serum oxytocin levels. Contrary to our hypothesis, it was observed that exogenous oxytocin administered during labor did not significantly affect either maternal serum oxytocin levels or umbilical cord blood oxytocin levels. This finding highlights the importance of preserving the natural process in stimulating the endogenous oxytocin system of the mother and fetus during synthetic oxytocin administration.

Considering the reciprocal nature of breastfeeding and mother-infant bonding, this study also took into account the newborn’s cord endogenous oxytocin concentrations in the early postpartum period (within the first minute after birth) when examining the mother’s breastfeeding behavior and maternal attachment levels. Although there is limited data on the direct effect of intrapartum administered synthetic oxytocin on fetal oxytocin levels, it is suggested that it may influence the newborn’s behaviors and early bonding processes after birth observed negative breastfeeding and primitive neonatal reflex outcomes in babies of mothers whose labor was induced or augmented with synthetic oxytocin [3,22,23]. They proposed that this might be due to synthetic oxytocin possibly crossing the placenta and the baby’s blood-brain barrier [22,23]. However, this situation requires a sufficient concentration of synthetic oxytocin to pass through two barriers—the placenta and the fetal blood-brain barrier—which are known to be reasonably impermeable to synthetic oxytocin [24]. The endogenous oxytocin levels of the newborns we examined as a result of low-dose synthetic oxytocin application support this hypothesis. Additionally, in our study, all mothers reported that they wanted to breastfeed their newborns, and the occurrence of breastfeeding within the first hour after birth indicates that synthetic oxytocin did not negatively affect the newborns’ sucking behavior.

The first 60–90 minutes after delivery are an important and optimal period for the early start of breastfeeding and the establishment of healthy maternal attachment. It was shown that the mothers who experienced breastfeeding immediately after birth exhibited more positive maternal behaviors [25,26].

The main question of the current study was whether synthetic oxytocin administration affects breastfeeding and maternal bonding. Breastfeeding and maternal bonding are processes in which the role of endogenous oxytocin is well established, but it is frequently reported that the relationship between the administration of synthetic oxytocin during labor and breastfeeding is negative [5,19,22]. A study reported that mothers who received synthetic oxytocin induction are more likely to have breastfeeding problems after birth [27]. In another one, breastfeeding was evaluated at the same time, as in the current study, and it was reported that mothers who received synthetic oxytocin at birth experienced a negative breastfeeding experience within the first hour after birth [28]. However, data from a recent study reported that there was no negative effect on oxytocin release during early breastfeeding in women who received only exogenous oxytocin at birth [29]. Some researchers have focused their hypotheses on maternal physiology, such as discussing the possibility that exposure to synthetic oxytocin during labor may desensitize the mother’s oxytocin receptors [10,30]. The rate of initiating breastfeeding within the first half hour postpartum was 56% in those receiving synthetic oxytocin, while this rate was 80% in those not receiving synthetic oxytocin [31]. The rate of initiating breastfeeding within the first hour after birth was found to be 76% versus 91%, respectively [28]. In this study, the mothers nursed their babies mean of 40–47 minutes, and these times were similar in both groups (p > 0.05). However, the time it took to hold their babies for the first time after birth was found to be significantly longer in those who received oxytocin during labor (41 ± 38 vs 27 ± 43 minutes, p < 0.05). This result may be interpreted that although the exogenous oxytocin was administered in the case group according to the low-dose regimen, its administration may have tired mothers. This may have led mothers to want to rest at first and not want to hold their babies immediately after giving birth. The mothers wanted to see their newborns immediately (in 3 minutes) after the first care was completed and started breastfeeding early. However, the mothers held their babies late compared to breastfeeding, possibly because the mothers had been supported by midwives and had started breastfeeding immediately after birth.

BAES is a scale developed to evaluate early breastfeeding, and we used this scale to assess the positive or negative effect of oxytocin administered during labor on the breastfeeding attitudes of mothers when they first started breastfeeding. BAES scores were found to be similar in both groups in the current study. This suggests that the administration of synthetic oxytocin during labor did not have any effect on early breastfeeding, since the measured serum oxytocin levels were similar in both groups. Similarly, it was reported that synthetic oxytocin administration did not influence early breastfeeding behavior after delivery [32]. Different results present in the literature and found in this study suggest that other variables, such as the presence of other interventions applied at delivery, affect breastfeeding attitude, supportive practices for initiating breastfeeding, and the desire to breastfeed.

Oxytocin plays an important role in the positive development and maintenance of maternal attachment, and is also associated with the relationship of mothers with their parents, friends, and environment [30,33]. The Maternal Attachment Scale (MAS) was used in this study to evaluate the relationship between oxytocin levels and maternal bonding between mother and baby, and MAS scores were found not to be statistically different between the groups. We would like to remind you that there was no difference in the measured serum oxytocin levels between the two groups. Although a similar study examining the relationship between oxytocin administration during labor and maternal attachment did not find a difference between the groups [34], there are also studies reporting that early postpartum oxytocin level affects the mothers’ caregiving behaviors and the MAS score [16,17]. Studies are showing that maternal-infant bonding is positively affected in those given exogenous oxytocin during labor [11], while there are also studies reporting that it is negatively affected [35]. These different results may be due to differences in the focus of each study and the applied methodology. In the current case-control study, the effect of synthetic oxytocin administration on maternal attachment was assessed by considering the serum oxytocin level measured in serum, while Edwars et al. measured oxytocin levels in saliva and assessed the relationship between mother-infant attachment using observational methods. The authors also noted that synthetic oxytocin was used to shorten labor duration, which may have influenced mother-infant bonding outcomes reported similar results to our study with a very large sample size (n = 19700) [11]. However, they only included mothers who were induced for labor (by synthetic oxytocin or otherwise) and investigated the effect of induction methods on negative maternal bonding. They excluded mothers whose labor started spontaneously. In addition, synthetic oxytocin applications for preventing, managing, or treating postpartum hemorrhage were not taken into account in the analyses [35].

The strength of this study is that the oxytocin level, which has a very short half-life (approximately 2–4 minutes), was measured from the maternal and umbilical blood samples taken immediately (within the first minute) after birth. Hence, the possible effects of other emotional and environmental variables on the breastfed could be eliminated.

The limitation of this study is that it examined only the relationship between serum oxytocin level, breastfeeding attitudes, and maternal attachment. However, previous birth and breastfeeding experiences may also affect the current oxytocin levels.

In conclusion, it was observed that intravenous synthetic oxytocin administered to initiate or strengthen uterine contractions during labor in delivery units did not affect the maternal and neonatal serum oxytocin levels and did not cause negative effects on early breastfeeding attitudes and maternal attachment.

## Supporting information

S1 File Dataset. (SAV)
